# In vivo evidence of exosome-mediated Aβ neurotoxicity

**DOI:** 10.1186/s40478-020-00981-y

**Published:** 2020-07-06

**Authors:** Ahmed Elsherbini, Haiyan Qin, Zhihui Zhu, Priyanka Tripathi, Simone M. Crivelli, Erhard Bieberich

**Affiliations:** Department of Physiology, University of Kentucky College of Medicine, 800 Rose Street Room MS519, Lexington, KY USA

**Keywords:** Astrocytes, Exosomes, Mitochondria, Ceramide, Amyloid

Alzheimer’s disease (AD) is the most prevalent form of dementia in elderly patients. Amyloid beta (Aβ) is one of the most studied contributors to AD pathology, however, the intracellular toxicity mechanism of Aβ is not clear yet. Recently, a wealth of publications have demonstrated ways by which exosomes could participate in the pathology of AD either in a protective manner or as a facilitator for plaque deposition and shuttling of misfolded protein [1, 3, 4]. In our recent publication [2], we showed that serum from the transgenic mouse model of familial AD (5xFAD) and AD patients as well as 5xFAD brain-derived exosomes contains exosomes that are associated with Aβ. The association with exosomes was shown to substantially enhance Aβ neurotoxicity in AD. When taken up by neurons in vitro, these Aβ-associated exosomes were transported to mitochondria, induced mitochondrial clustering, and upregulated the fission protein Drp-1. Aβ-associated exosomes mediated binding of Aβ to voltage-dependent anion channel 1 (VDAC1) and subsequently, caspase activation. Aβ-associated exosomes also induced neurite fragmentation and neuronal cell death. However, despite solid and convincing results, our study lacked the in vivo component. In this addendum, we aim to augment the significance of our previous work by adding in vivo data that confirms our previous results.

For these experiments, brain-derived exosome isolation was performed as previously described [1]. Briefly, donor 5xFAD mice (9 months old, one male and one female) were anesthetized with isoflurane then perfused with cold 1x PBS to remove exosomes from the brain blood circulation. After collecting the brains, they were washed with 1x PBS and cut into eight sagittal slices. Brain slices were then transferred to gentleMACS C tubes and mixed with enzymatic dissociation buffer. Program 37C_ABDK_01 was used for gentleMACS Octo Dissociator with Heaters. Samples were resuspended and applied to a MACS SmartStrainer (70 μm) placed on a 50 mL tube. Ten milliliters of cold D-PBS were applied onto the MACS SmartStrainer (70 μm). Cell suspensions were centrifuged at 300×g for 10 min at 4 °C, supernatants were carefully transferred to a fresh tube and mixed with cocktail protease inhibitor. Supernatants were centrifuged at 2000×g for 10 min followed by 10,000×g for 30–40 min then passed through a 0.45 μm filter. Afterwards, samples were subjected to Exoeasy exosome isolation protocol as described before [2]. Exosomes were then labeled with the lipid binding dye Vybrant Cm DiI. Ten microliter of exosomes were then injected into the brain of 3 weeks old wild type (WT) mice (*n* = 8, four mice per group) using stereotactic injection. The injection site coordinates (from bregma) Anterior Posterior (AP) – -2.12 mm, Medial Lateral (ML) + 1.6 mm, Dorsal Ventral (DV) 2 mm from skull). Forty-eight hours post-injection, mice were sacrificed, and brains were fixed in 4% PFA, followed by 40% sucrose in PBS and the O.C.T medium before freezing for cryo-sectioning. Eight sections were obtained from each mouse around site of injection.

Here we report similar findings in vivo as we found in tissue culture experiments. Firstly, labeled 5xFAD exosomes are taken up by neurons in WT mouse brain as denoted by the presence of Vybrant Cm DiI labeled exosomes inside Neurotrace positive cells (Fig. 1c). Interestingly, WT exosomes were taken up to a lesser extent (Fig. 1b). Next, we labeled brain sections for both the mitochondrial protein Tom-20 and Neurotrace to investigate the shuttling of exosomes to mitochondria as determined in our in vitro experiments. As expected, we found solid colocalization between 5xFAD-derived and labeled exosomes and Tom-20 inside neurons (Fig. 2a), which was also detectable with WT exosomes, but to a lesser extent. In order to investigate the subsequent mechanism of this interaction between mitochondria and exosomes, we performed proximity ligation assay using antibodies against Aβ and Voltage-dependent anion channel 1 (VDAC1), the main ADP/ATP transporter in the outer mitochondrial membrane. We found positive signals (denoted by green dots) inside neurons of brains injected with 5xFAD exosomes, but not in the brains injected with WT exosomes (Fig. 3a, b), suggesting that Aβ was shuttled via these exosomes to neurons and associated with mitochondrial VDAC1. Moreover, the association of 5xFAD exosomes and Aβ lead to caspase activation in neurons as demonstrated by the presence of cleaved-caspase signal in neurons that took up 5xFAD exosomes, which was not observed when injecting WT exosomes (Fig. 3c, d). In conjunction with our previous data, these results demonstrate the relevance of exosomes in Aβ induced neurotoxicity in vivo, suggesting that disruption of Aβ association to exosomes offers a new therapeutic approach to AD.

**Fig. 1:**
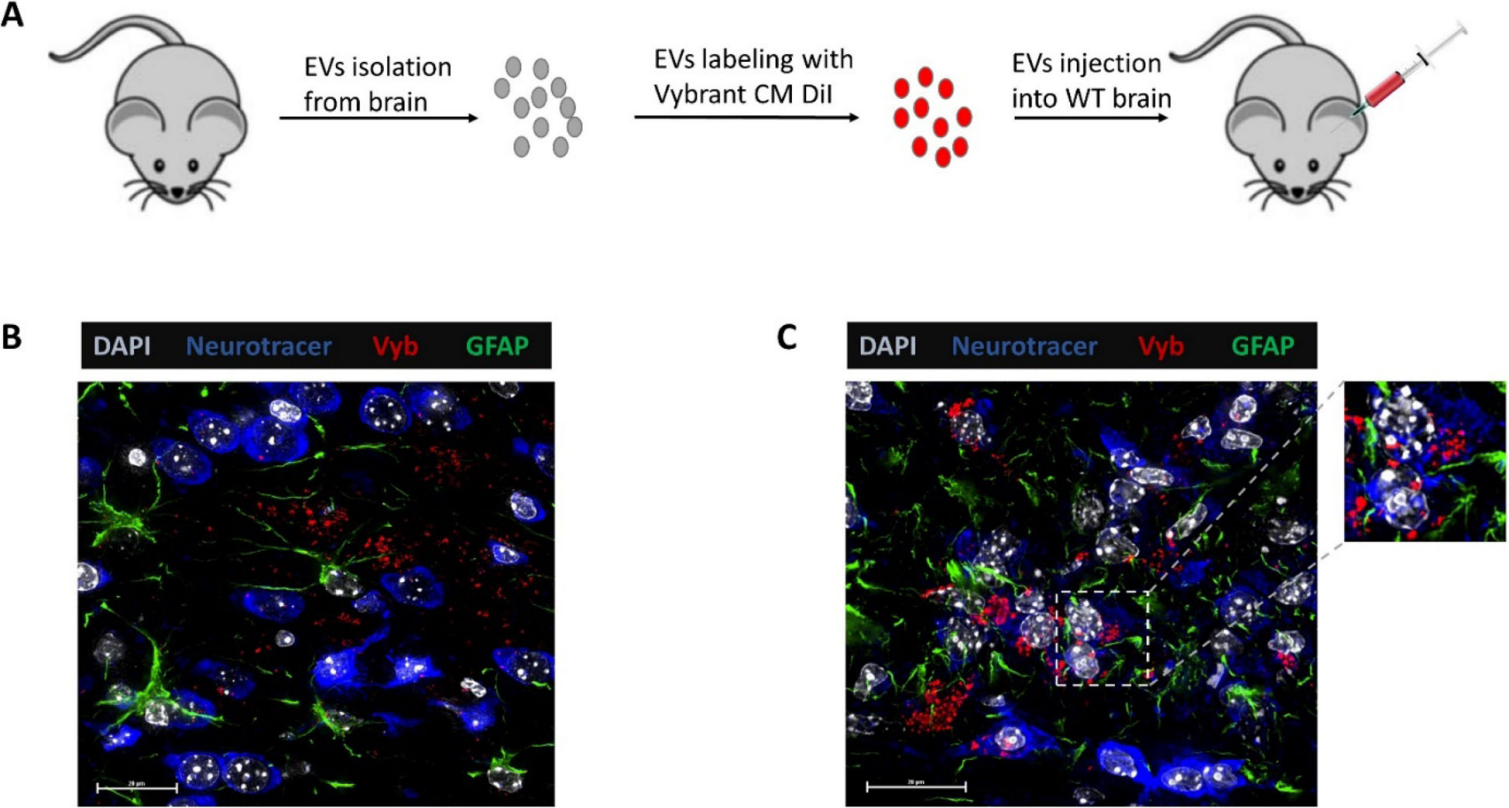
5xFAD exosomes are taken up by WT neurons in vivo. (**a**) A schematic diagram of the experimental design. Exosomes isolated from brains of 5xFAD mice were first labeled with lipid-binding dye Vybrant CM DiI before being injected intracranially into wild type (WT) mice. Forty-eight hours post-injection, mice were sacrificed, and brains were collected and prepared for cryo-sectioning. (**b**, **c**) Representative immunocytochemistry images of sections of brains injected with (**b**) WT exosomes and (**c**) 5xFAD exosomes showing that FAD exosomes are internalized by WT neurons, insert is a higher magnification of the selected ROI

**Fig. 2:**
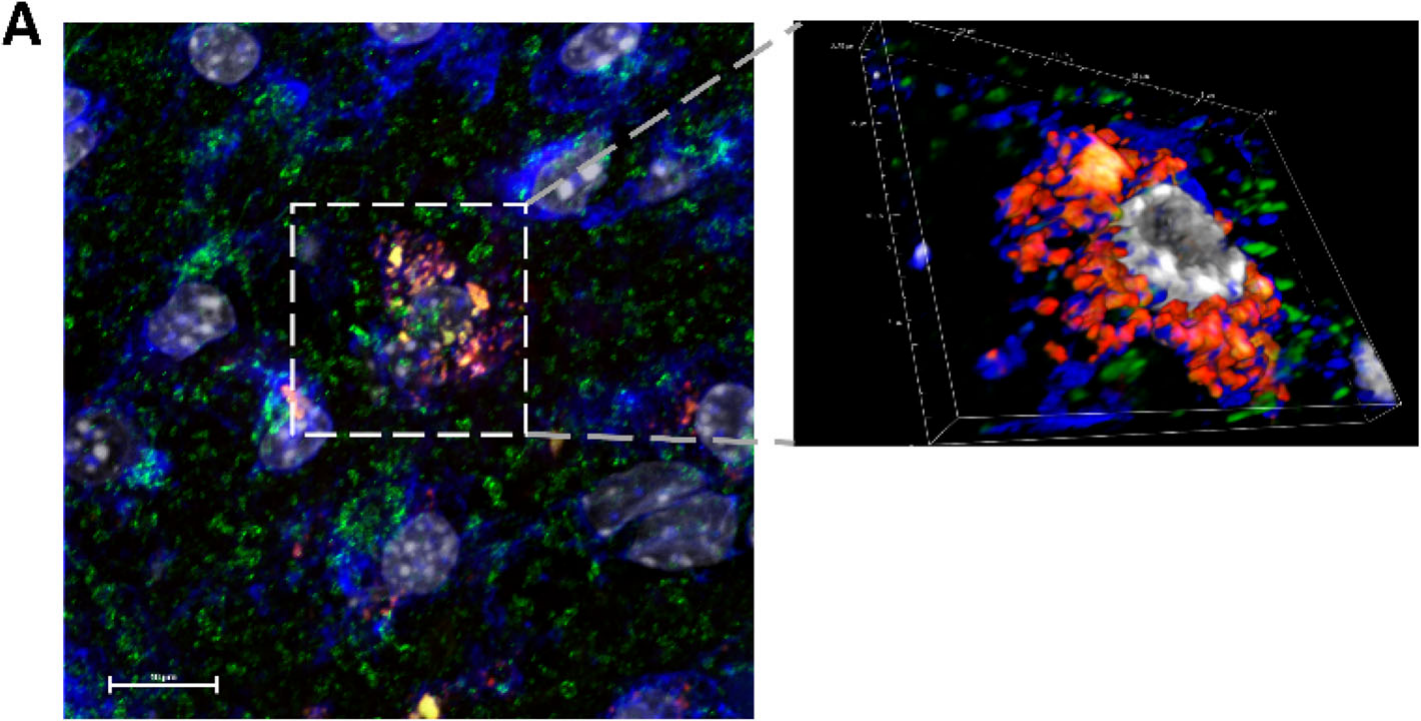
5xFAD exosomes associate with neuronal mitochondria: (**a**) Representative confocal images of brain section showing that FAD exosomes (red) are colocalized with Tom-20 (green) inside neurons (blue). Insert is a 3D rendering of the selected ROI

**Fig. 3:**
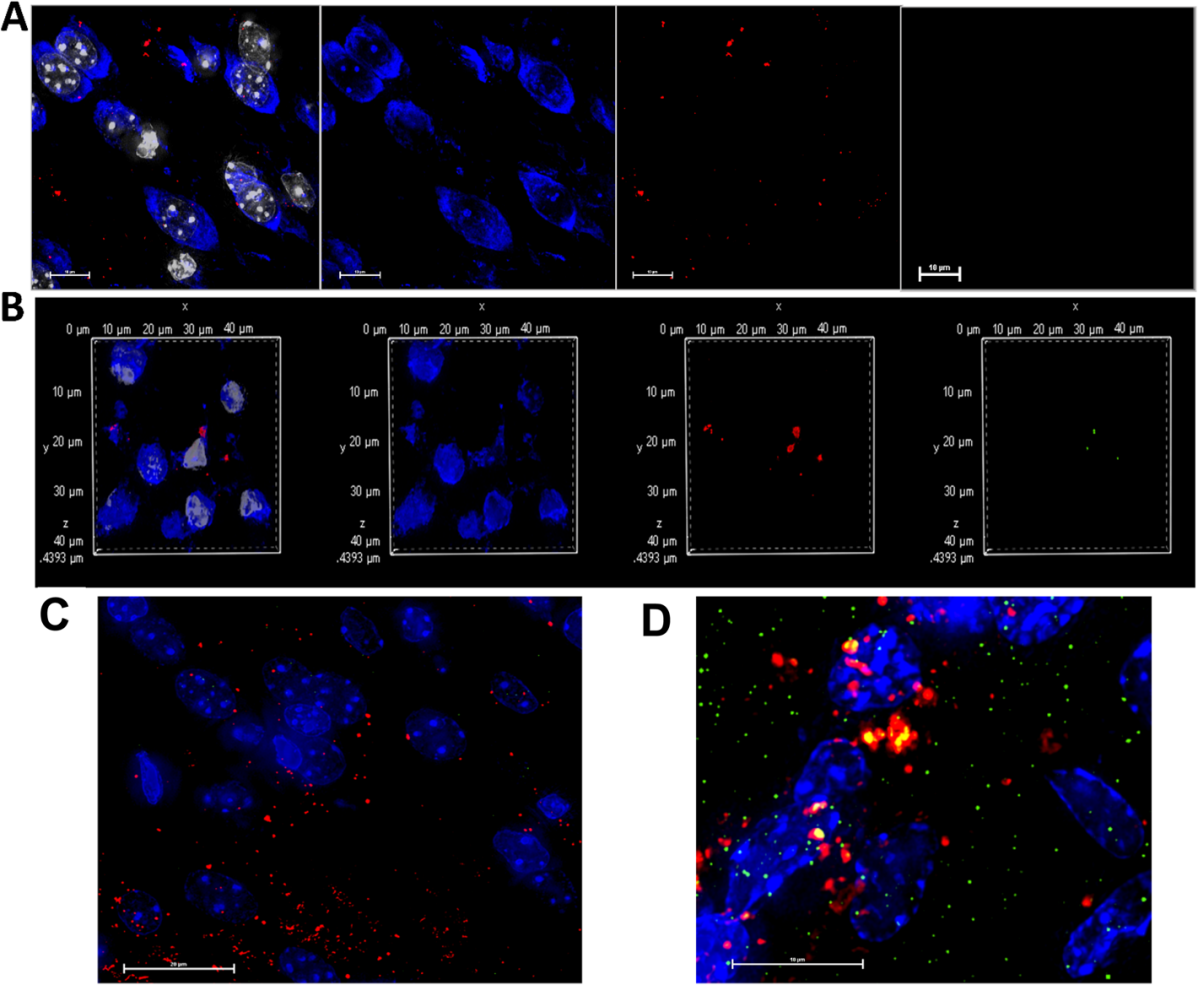
5xFAD exosomes associate with neuronal mitochondria VDAC1 leading to caspase activation. Representative photomicrographs of (**a**) WT exosomes and **(b**) 5xFAD exosomes injected in brains of WT mice showing PLA complex formation (green) inside neurons (blue) in brains injected with 5xFAD exosomes. (**c**) WT exosomes injection showing no caspase activation, while **(d**) shows 5xFAD exosome- injected brains showing positive cleaved caspase-3 signals (green) colocalized with labeled exosomes (red)

## Data Availability

All data generated or analysed during this study are included in this published article.
